# Current Concepts Underlying Benefits of Exercise Training in Congestive Heart Failure Patients

**DOI:** 10.2174/157340310791162640

**Published:** 2010-05

**Authors:** Maqsood Elahi, Mohsin Mahmood, Ahmad Shahbaz, Naveed Malick, Jawad Sajid, Sanjay Asopa, Bashir M. Matata

**Affiliations:** 1 Wessex Cardiothoracic Centre, General/ BUPA, Chalybeate Close, Southampton, SO16 6UY, United Kingdom; 2 Department of Medicine, Mayo Hospital, King Edward Medical University, Lahore, Pakistan; 3 Department of Cardiovascular Surgery, Punjab Institute of Cardiology, Ghaus-ul-Azam Road, Lahore, Pakistan; 4 Department of Medicine and Elderly Care, General Hospital, Tremona Road, Southampton, SO16 6YD, United Kingdom; 5 The Cardiothoracic Centre, Liverpool NHS Trust, Thomas Drive, Liverpool, L14 3PE, UK

**Keywords:** Heart failure, exercise training, proinflammatory cytokines, nitric oxide, myocardial function, ventilatory function, quality of life.

## Abstract

The pathophysiology of several conditions including heart failure is partly attributable to a failure of the cell energy metabolism. Studies have shown that exercise training (ET) improves quality of life (QOL) and is beneficial in terms of reduction of symptoms, mortality and duration of hospitalization. Increasingly, ET is now achieving acceptance as complimentary therapy in addition to routine clinical practice in patients with chronic heart failure (CHF). However, the mechanisms underlying the beneficial effects of ET are far less understood and need further evaluation. Evidence suggests that while CHF induces generalized metabolic energy depletion, ET largely enhances the overall function of the heart muscle. Hence, research efforts are now aiming to uncover why ET is beneficial as a complimentary treatment of CHF in the context of improving endothelial function and coronary perfusion, decreasing peripheral resistance, induction of cardiac and skeletal muscle cells remodeling, increasing oxygen uptake, substrate oxidation, and resistance to fatigue. Here we discuss the current evidence that suggest that there are beneficial effects of ET on cardiac and skeletal muscle cells oxidative metabolism and intracellular energy transfer in patients with CHF.

## INTRODUCTION

Chronic heart failure (CHF) is a common condition with poor prognosis [1]. CHF is characterized by the inability of heart muscle to meet tissue energy demand that results in symptoms of fatigue or dyspnoea initially on exertion and then later on progressing to even at rest [1-2]. Actually, ventricular dysfunction is usually the major component of the CHF syndrome, which occurs in conjunction with skeletal muscle structural and functional abnormalities [3-4]. There is also a perception that the underlying mechanisms are largely dependent on oxidative energy production and control of energy fluxes [3-4]. Likewise, dyspnoea appears to be more closely related to increased ventilatory effort than pulmonary congestion, a feature that varies between patients [5-6].

The current perception is that CHF is a paradigm of energetic failure affecting cardiac and skeletal muscles, progressing to contractile failure, worsening physical disability, and eventually death. This radical shift in concept is supported by the change from treatment with sympathomimetic drugs to beta blockers. Given that in late 1980’s decreased left ventricular systolic function, cardiac enlargement and heart failure were considered absolute or relative contraindication to ET, it is now increasingly apparent that the lack of physical activity leads to skeletal muscle atrophy, exercise intolerance, venous thrombosis & pulmonary embolism [7]. Therefore, the use of ET in the treatment of CHF is being readdressed in the context of improving endothelial function, coronary perfusion, decreasing peri-pheral resistance, inducing cardiac and skeletal muscle cell remodeling, increased oxygen uptake and resistance to fatigue [8].

It is unclear however, as to what extent the observed beneficial effects of ET in CHF patients are based on cardiac improvement. Whether exercise therapy improves cardiac and skeletal muscle cells energy metabolism of patients with CHF is unknown. Here, we review the role of exercise therapy in the management of patients with CHF. In this review, focus is on the different modes of ET, duration, exercise dynamics in the context of cardiac rehabilitation and heart failure. Finally, we also present current knowledge on the effects of ET on changes to energy metabolism for chronic heart failure patients.

## CHF AND UNDERLYING MECHANISMS OF PROPAGATION

### Deranged Energy Metabolism 

In normal circumstances, heart muscle maintains blood circulation in states of both rest and high peripheral demand a process that requires the transfer of vast amounts of energy between various compartments. The energy transfer process is usually permanent, adaptable, rapidly regulated, and highly efficientt. In contrast, the failing heart has been described as energy starved [9]. In the early stages of heart failure, its oxidative capacity decreases and the energy transfer and utilization get impaired [10]. Heart muscle fibers mainly rely on mobilisable energy sources to develop strong and long-term contractile activity via oxidative phosphorylation. Because of limited cellular metabolic reserves (mainly phosphocreatine (PCr) and glycogen), fatigable muscle exhibit delayed recovery of their energy reserves (through anaerobic glycolysis and less importantly mitochondrial oxidation). The metabolic specificity of muscle types generates fiber-type specific substrate utilization. In general, oxidative fibers and cardiac muscle oxidize fatty acids and lactate, while glycolytic fibers mainly use glucose as substrate [11]. Creatine kinase (CK) and other phosphotransfer kinases (like myokinase) participate in this energy shuttling within cardiac and skeletal muscle cells [12-13] and utilize myosin and sarcoplasmic reticulum ATPases (SERCA). Energy is channeled directly between mitochondria and the sarcoplasmic reticulum (SR) or myofibrils in slow skeletal and cardiac muscle to produce compartments of the adenine nucleotides [14].

Another recent development in the understanding of energy metabolism has been in the area of the transcriptional regulation and signaling pathways involved in the maintenance of energy homeostasis and mitochondrial biogenesis for the failing heart [15]. The mitochondrial transcription factor (mtTFA) is encoded by the nuclear genome and activates the transcription and replication of mitochondrial DNA. The expression of mfTFA is controlled by the nuclear respiratory factors (NRFs) that additionally stimulate the expression of numerous nuclear-encoded mitochondrial proteins under the control of transcriptional co-activator PGC-1α (transcriptional co-activator of peroxisome-proliferator-activated-receptor (PPAR) gamma) [16-19] PGC-1α coordinates mitochondrial protein expression and substrate utilization by co-activating PPARα that regulates fatty acid oxidation and transcription of nuclear and mitochondria-encoded proteins. Finally, pressure overload-induced hypertrophy results in deactivation of PPARα and subsequently dysregulation of fatty acid oxidation enzyme gene expression [20]. However, which signal triggers the drop in creatine kinase in heart failure, remains to be established.

### Inflammatory Process 

Evidence suggests a state of heightened immune activation in CHF. [21-22] Pro-inflammatory cytokines interleukin-2 (IL-2), Interleukin-6 (IL-6), and tumour necrosis factor-α (TNF-α) are found in higher concentrations in the circulating blood of ischaemic heart disease and CHF patients compared with normal subjects [23]. Studies have demonstrated that TNF-α can depress myocardial contractility and induce cardiomyopathy on its own [24]. Cytokines including TNF-α promote over expression of inducible nitric oxide synthase [25], cause elevated levels of intracellular nitric oxide that are sufficient to depress oxidative capacity and myocardial function. Moreover cytokines including TNF-α, are stimulators of apoptotic cell death [21-22]. The basis for the heightened immune response in heart failure is yet to be elucidated, however possible mechanisms postulated include tissue hypoxia [22], myocardial oxidative stress production and endo-toxin mediated stimulation [26-27] as summarised in Fig. (**1**).

## RELEVANCE OF EXERCISE TRAINING IN CHF

“Respiratory muscle deoxygenation” implies that ventilation/perfusion mismatching is caused by the diaphragm and intercostal muscle metabolic impairments. While these exist, ventilation/perfusion mismatching is caused largely by an impaired cardiac output response to exercise [7]. Evidence demonstrates that ET is associated with lowering concentration of inflammatory markers including C-reactive protein (CRP), TNF-α, and Serum amyloid A (SAA) in patients’ with CHF [28-29]. Mechanisms responsible for the anti-inflammatory effects of ET are not fully elucidated; however fat loss, [28] changes in CRP concentration, [28] increased anti-inflammatory cytokines in the peripheral blood mononuclear cells, [30] and increased antioxidant capacity in skeletal muscle [31] contribute to such effects.

ET in CHF works by many factors and trials demonstrate that peak oxygen uptake (VO_2_) is reduced in CHF patients [7]. The rationale for the reduced VO_2_ in HF includes a lower exercise cardiac output response, reduced nutrient blood flow to the active skeletal muscles, along with skeletal muscle abnormalities such as a reduced percentage of type-I fibres, weakened oxidative capacity and a reduction in the capillary density [7]. Skeletal muscle biopsy studies have established defects in oxidative and glycolytic enzymes that explain the metabolic abnormalities [32-33]. These histologic and metabolic disorders are in part similar to those seen in severe deconditioning and can be at least in part prevented by regular ET [34]. These metabolic abnormalities explain changes such as early fatigability and reduction in maximal strength that are very common in heart failure [35].

However, due to concerns regarding accuracy and reliability of ET, the (VO_2_ _max_.) is now more commonly accepted as a direct measure of cardio-respiratory endurance during exercise of increasing intensity [1,36-38]. Virtually all studies have shown a strong correlation between total body VO_2_ _max_ and survival in CHF [38]. Nevertheless, the majority of trials have accessed the benefits of ET in patients’ with CHF not only by exercise duration but more importantly by peak VO_2 _[1,29-40].

Improvement in peak exercise capacity and an improved ability to tolerate sub maximal exercise represent clinically important and expected outcomes in stable patients with mild to moderate heart failure. Recent work of follow-up in over 150 CHF patients demonstrated a significant increase in both peak VO_2_ and muscle strength, but no improvement in 6-minute walk distance [28]. The possible mechanisms responsible for the ET induced increase in peak VO_2_ represent an assimilation of several peripheral factors like reduced vasoconstriction, improved oxygen extraction and metabolic function at the active skeletal muscles^1^. Time to ventilatory derived anaerobic threshold and oxygen con-sumption at this threshold are also increased with training, suggesting delayed reliance on anaerobic pathways for energy production [1, 18].

## EXERCISE TRAINING AND IMPROVEMENT IN MYOCARDIAL FUNCTION

The effects of exercise training on both LV systolic and diastolic function are so far not conclusive and require larger trials with longer follow-up. Belardinelli and co-workers reported an improvement in diastolic filling rate that was related with an increase in cardiac index at peak exercise [36]. Similarly, ET duration is an important factor in implementing the improvement in cardiac function [40]. This suggests that long term ET is required to achieve central hemodynamic changes at any given workload. The severity of heart failure and the nature of training also modulate the effects of ET. Bank and associates demonstrated that improvement in endothelium-dependent vasodilatation after forearm exercise training is less readily obtained in patients’ with heart failure than in the control subjects [41].

ELVD-CHF, a multi-centre randomised controlled trial involving 90 patients with CHF demonstrated that long term moderate ET in stable CHF patients with severe LV systolic dysfunction has an anti-remodelling effect on LV volumes with significant improvement in ejection fraction [42]. In addition, ET in patients’ with post-infarction LV dysfunction does not have any harmful effect on LV volumes; rather it attenuates the remodelling process^41^. Importantly emerging investigations [17, 43-44] have shown that the infiltration/activation of monocytes contribute to the development of remodelling and heart failure. ET intervenes at various stages of the inflammatory process in patients’ with CHF and exerts a favourable control of remodelling by reducing the major circulating pro-inflammatory cytokines and their soluble receptor.

Growing evidence supports the notion that ET, often combined with endurance exercise, is efficient in patients with CHF [45]. In two small separate cross-over studies, Maiorana and co-workers [46-47] demonstrated that CT improved aerobic capacity, muscle strength and peripheral endothelial function. These data were later confirmed in a larger study involving a total of 39 CHF patients [48]. Others have shown increased skeletal muscle mitochondrial ATP production rate and higher capillary density, as well as anti-inflammatory effects [49-50].

## EXERCISE TRAINING AND VENTILATORY FUNCTION

The early onset of dyspnoea in CHF patients’ is mainly caused by altered ventilatory response to exercise due to increased dead space ventilation resulting from pulmonary congestion. Presence of cardiac insufficiency in CHF patients’ induces skeletal and respiratory muscle myopathy reflected by a peripheral catabolic state as evidenced by increased lactate production and acidosis [51]. The lactic acidosis (mediated by the metabolites) directly or indirectly may stimulate hyperventilation by acting on reflex control at rest and on exercise, leading to a reduction in arterial PCO_2_. Similarly, stimulation of chemoreceptors may show oscillation in respiration, heart rate and blood pressure modulated by cerebral circulation [51]. Lactic acidosis would be present at rest only in extreme circumstances.

Although the origin of dyspnoea is multifactorial, the increased ventilation in CHF plays an important role and is characterised by both rapid and shallow respiration. However measurement of ventilatory exchange during exercise shows that the ratio of dead space/tidal volume (VD/VT) is increased suggesting that ventilation/perfusion defects develop during exercise [1]. Furthermore early activation of muscle ergoreflex contributes to an excessive increase in sympathetic activation and exercise hyper-ventilation [51]. ET largely attenuates the abnormal venti-latory response to exercise in CHF patients by a delay in blood lactate accumulation, better ventilation/perfusion matching and attenuation of the ergoreflex activation [1, 51].

## EXERCISE TRAINING AND IMPROVEMENT OF SKELETAL MUSCLE FUNCTION

Histological and metabolic abnormalities of muscle cells, and skeletal muscle wasting have been described in CHF patients [52]. Anaerobic metabolism is found to occur early in patients with HF undertaking ET. This was found to be independent to the reduced blood flow to the muscle in this subset of patients [32]. Duscha *et al*, suggested that in patients with HF, an increase in capillary density would be a favourable adaptation. Moreover, the authors noticed a significant reduction in the microvascular density in patients’ with CHF without major differences in other histologic and biochemical aerobic markers [53]. The skeletal muscle dysfunction in heart failure is due to atrophy of muscle fibre, a relative decrease in oxidative type-I fibres, loss of microvasculature, decrease in enzymes of oxidative metabolism, and attenuation of mitochondrial surface density and size [3, 30]. ET has been shown in part to correct the skeletal muscle abnormalities in these patients. Several trials using a limb training model have documented improvements in muscle functions as measured by muscle strength, exercise time, phosphocreatinine re-synthesis and depletion rates, adenosine diphosphate concentration and intracellular pH [7,54].

## EXERCISE TRAINING AND IMPROVEMENT OF ENDOTHELIAL FUNCTION

CHF patients have a subnormal peripheral blood flow response to exercise. This is not only due to reduced cardiac output but also to an abnormal peripheral vasodilatory response. The abnormal peripheral vasodilatory response to ischaemic challenge could be due to a variety of stimuli including isotonic and isometric exercise, adrenergic blockade, and ischemia and arterial vasodilatation [51-55]. An additional mechanism suggested involves vascular stiffness secondary to increased vascular sodium content as evidenced by the fact that the capillary basement membrane may be thickened in HF and that the vascular responsiveness is partially improved by diuretic therapy [7, 54]. Endothelium dependent dilatation of the vasculature is impaired in HF as demonstrated by the reduction in the release of nitric oxide in response to acetylcholine [7, 55]. The release of nitric oxide, an important mediator of the flow-dependent vasodilatation is stimulated by exercise in healthy individuals but seems to be attenuated in CHF patients. This contributes to reduction in peripheral vasodilatation and thus tissue perfusion. This has been supported by the fact that blockade of nitric oxide synthesis reduces the flow to exercising muscle and L-arginine (nitric oxide precursor) supplementation improves the abnormal vasodilatation in response to ischemia in patients’ with HF [23]. 

Exercise training also enhances oxidative capacity of the skeletal muscle and corrects endothelial dysfunction of the skeletal musculature in ischemic and dilated cardiomyopathy [23]. This contributes to the reduction of peripheral resistance and improvement in stroke volume. This in part could explain the improvement of myocardial perfusion observed even in the absence of changes in coronary artery diameter [43]. Although it is well known that ET improves vasodilator function it is not clear whether other markers such as reduced vasoconstrictor tone and improved large vessel function via enhanced endothelial vasodilator function contributes to this.

## EXERCISE TRAINING AND ENDOTHELIAL PROGENITOR CELLS (EPC)

There is relatively very little information available in the literature on the effect of ET on circulating EPC in patient with CHF. Single centred studies with small number of patients have shown that intensive and moderate ET for long periods increased circulating levels of EPC in normal subjects [56]. Increased numbers of circulating endothelial progenitor cells (EPC) are associated with improved vascular function. Endothelial progenitor cells (EPCs) were experimentally shown to incorporate into sites of neovascularization and home to sites of endothelial denudation [56]. Increased oxygen demand may represent an important beneficial outcome of physical exercise, supporting the notion that increased numbers of EPC correlate with cardiovascular health. 

Dimmeler’s group previously reported the inverse relationship between the patients with coronary artery disease (CAD) and circulating EPC levels and function [57]. Furthermore, they also studied whether this impairment extends to bone marrow–derived mononuclear cells (BM-MNCs) in patients with chronic ischemic cardiomyopathy (ICMP). They found a significantly fewer colony-forming capacity of BM-MNCs from patients with ICMP, along with reduced migratory response to challenge *in vitro*. The authors further reported reduced neovascularization capacity despite similar contents of hematopoietic stem cells *in vivo *[58]. Similarly Goette *et al* reported that moderate-to-severe CHF is not associated with elevated EPC levels in the systemic circulation. They also could not demonstrate a measurable cardiac uptake of EPC in this cohort [59].

## EXERCISE TRAINING AND IMPROVEMENT OF QUALITY OF LIFE

CHF patients experience progressive decline in the quality of life (QOL), limiting their routine daily activity due to the early onset of dyspnoea and fatigue. In addition depression and isolation are common in these patients. Even patients with stable heart failure suffer reduced QOL as symptoms relief is not complete [1].

Only a few studies of heart failure patients participating in exercise training programme have included the QOL questionnaire in their study protocol. In addition the QOL measurement tools vary and the results are inconsistent. The majority of the trials used “Minnesota Living with Heart Failure questionnaire” that assesses disease-specific health-related QOL and its effects on daily life [1, 60]. However few trials used dyspnoea-fatigue index, [8, 61] a self administered 5-point Likert scale instrument measuring magnitude of each component; the specific dyspnoea-provoking task, the pace with which the task is performed and the patient’s general functional capacity [8]. Majority of the trials that measured QOL in HF patients ranged in sample size from 25 to 99, undergoing 3 to 12 months of exercise training. Majority of the trials show a significant improvement in QOL and improvement in exercise capacity, [1,33] with few trials demonstrating no improvement in QOL [62]. The earlier mentioned difference in outcome could be explained on the basis of different protocols and variable tools for the measurement of QOL.

It is still not clear, whether ET in HF has any long term effects on QOL as no strong correlation exists between measures of health related QOL and central hemodynamic abnormalities and exercise intolerance [7]. Nevertheless, ET is proving to be an effective treatment for improving QOL as patients become more tolerant of exertion; and experience less fatigue and dyspnoea and become comfortable performing tasks of daily living.

## EXERCISE TRAINING AND SURVIVAL

Currently there is no published large randomised controlled trial studying the long term clinical effectiveness of ET in CHF patients. Recently published literature comprises of mostly single centred experience containing small number of patients with short follow up. Belardinelli & co-workers analysed 99 patients with a mean follow up of 3.4 yrs. In their study, patients in the exercise training group had fewer deaths (all deaths were cardiac deaths) (P=0.01); fewer hospitalizations for chronic heart failure (P=0.02); fewer cardiac events (P=0.006); increased peak oxygen consumption per unit time (P<0.001) and thallium uptake (P<0.001); and improved qualify of life at 2, 14, and 26 months [29].

Whellan & colleagues identified 70 patients in their database with HF who participated in Cardiac Rehabilitation (CR) program. Participation in CR> 6 sessions was associated with significantly improved survival (*P *< .0001). The authors suggested a dose response with improved survival benefit for patients with CHF participating in cardiac rehabilitation [63]. 

Tenenbaum *et al* followed 42 CHF patients for over 3 yrs and reported increased survival benefits in patients that continued ET for 3 yrs compared to those who stopped after 1.6 yrs [64]. Smart and Marwick in 2004 identified a total of 81 studies in a meta-analysis (30 were randomized controlled trials, five nonrandomized controlled trials, nine randomized crossover trials, and 37 longitudinal cohort studies) and reported that mortality was not directly related to 60,000 patient-hours of exercise training. During the training and follow-up periods of the randomized controlled trials, there were 56 combined (deaths or adverse events) events in the exercise groups and 75 combined events in the control groups (odds ratio [OR] = 0.98; 95% confidence interval [CI]: 0.61 to 1.32; *P* = 0.60). During this same period, 26 exercising and 41 non-exercising subjects died (OR = 0.71; 95% CI: 0.37 to 1.02; *P* = 0.06). The authors suggested that ET is safe and effective in patients with heart failure. The risk of adverse events may be reduced, but further studies are required to determine whether there are any survival benefits [64].

## SUMMARY

As discussed earlier, cardiac failure itself is a syndrome of circulatory failure, secondary to ventricular dysfunction. This primarily ventricular dysfunction is followed by a variety of neurohumoral, peripheral circulatory, skeletal muscle, and respiratory adaptations which determine the syndrome's clinical presentation and prognosis more than the primary ventricular dysfunction itself. Traditionally, avoidance of exercise was thus advocated in all forms and stages of heart failure [65]. However, there is now evidence that inactivity leads to a further deterioration of remaining functional capacity. Published data so far on one hand is difficult to interpret with regard to the benefits of exercise duration and intensity on the outcome in chronic heart failure patients. Yet, more recently it has been shown that selected patients with compensated stable CHF can safely follow a training programme, thereby improving exercise tolerance and functional status [66-67]. 

Poor left ventricular function is not necessarily synonymous with CHF, which is characterized by reduced tissue oxygen supply. The best method of evaluating the disease state of a patient with a compromised heart is cardiopulmonary exercise testing, that is, the measurement of oxygen consumption (VO_2_ in ml/kg/min) during exercise. With ET, the observed improvements are sometimes small, but may have a major impact on quality of life in CHF patients. The difference after training is reflected in the patient's ability to cope with the physical demands of day to day activity, thus preventing the need for continuous bed rest and the loss of independence which often results in the need for chronic care.

The interpretation of reported results of dynamic exercise training in CHF is frequently complicated by the lack of sufficient power and the non-randomized trial design. Moreover, the incorporation of home-based activities and the use of unsophisticated tools for resistance training, such as elastic rubber bands, could lead to less strict adherence to prescribed activity. This set-up allowed close assessment of exercise intensity and work-volume and the early detection of adverse effects. Despite the fact that a cautious approach is still advocated in official recommendations on exercise training in CHF patients, scientifically correct evaluation of the safety of CT might be able to shift paradigms in the near future. Sustained isometric weightlifting at high intensity (80 to 90% of 1RM) and its possible detrimental effects are very distinct from dynamic resistive exercise prescription in cardiovascular patients. Several investigators have shown that the acute haemodynamic responses during resistance exercise in CHF patients were similar or lower than for aerobic exercise of comparable intensity.

Contraindications for CHF patients participating in such ET programme are the same as for patients with coronary artery disease [66]. While observing these contraindications, the studies reviewed showed no major complications during or shortly after exercise. By and large, all patients on optimal medical therapy for CHF and with stable heart failure should be advocated exercise training as this will compliment medical therapy. However, patients with acute CHF, structural valvular pathology and unstable coronary artery disease should be medically managed and optimized prior to embarking on exercise training. Patients with NYHA class IV symptoms should be optimized and class downgraded medical therapy. However, in patients with stable class IV symptoms trial of ET under careful supervision and monitoring particularly during the initial training period is important. Telemetry monitoring during these early sessions is also recommended in such a case.

Further research is thus necessary to confirm the role of ET as a treatment for patients with CHF, and to determine the most appropriate intensity, mode, and duration of training. Training can comprise overall endurance training monitored by heart rate, interval training based on percentage of maximum exercise level, or strength training. It has been suggested [67-68] that muscle groups can be trained simultaneously or consecutively. For training to be effective, the intensity should be sufficiently high, and the frequency should be at least three times a week. Initial training, lasting for at least six weeks, should be followed by a maintenance training regimen, since all improvements are reversible. An active lifestyle should be promoted.

In conclusion, exercise training in CHF patients has some merits and as discussed in this review, the changes induced by exercise at molecular level are well documented. However, how these changes translate into symptom improvement and QOL transformation is still not clear; in part due to lack of standardisation of the different ET protocols used. But before such patients can participate in a training programme, it is important to clarify the pathophysiology of their heart failure and thus identify their limiting factors. Finally the patient's individual profile requires definition: the training programme must be tailored to the patient's specific limitations and desired level of activity. Nevertheless, with the existing pool of evidence ET may safely be advocated to patients by health professionals keeping in view of their HF pathophysiology; whilst awaiting results from more robust large randomised studies. 

## Figures and Tables

**Fig. (1). Suggested mechanisms underlying the progression of heart failure.**
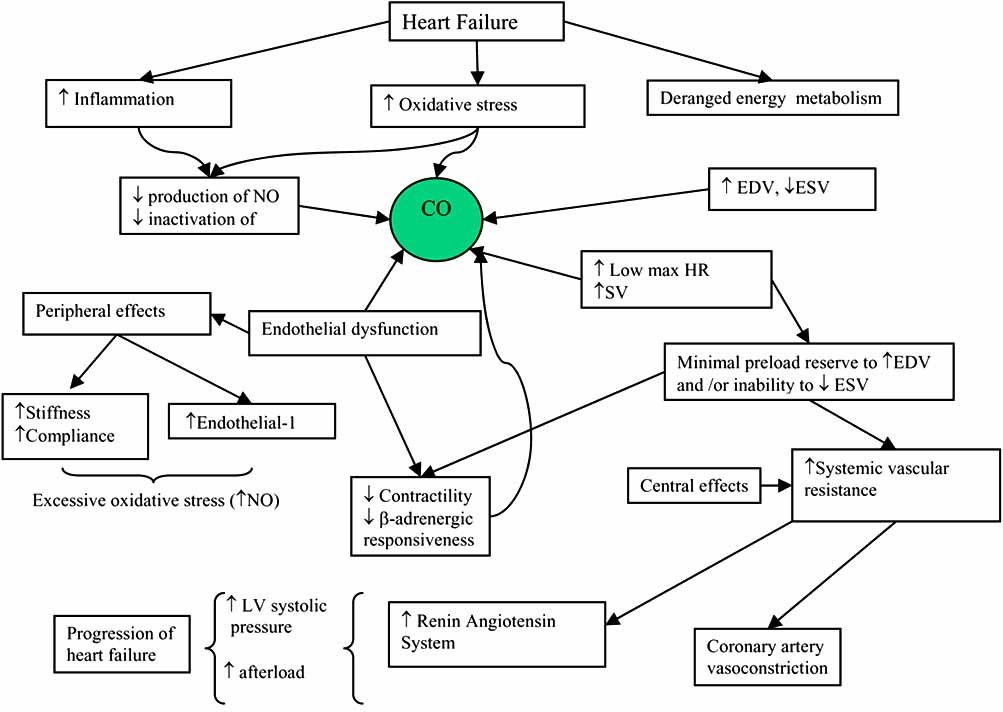
KEY: CO= cardiac output, HR= heart rate, EDV= end diastolic volume, ESV= end systolic volume, NO= nitric oxide, SV= stroke volume, LV= left ventricle.
